# Weight Gains Across Adulthood—The ELSA‐Brasil Study

**DOI:** 10.1111/cob.70095

**Published:** 2026-07-07

**Authors:** Scheine L. Canhada, Paula A. Bracco, Maria de Jesus Fonseca, Maria Del Carmen Bisi Molina, Maria Inês Schmidt, Bruce B. Duncan

**Affiliations:** ^1^ Postgraduate Program in Epidemiology, Universidade Federal do Rio Grande do Sul Porto Alegre RS Brazil; ^2^ National School of Public Health, Fundação Oswaldo Cruz Rio de Janeiro RJ Brazil; ^3^ Postgraduate Program in Nutrition and Health, Universidade Federal do Espírito Santo Vitória ES Brazil

**Keywords:** adults, epidemiology, obesity, weight gain

## Abstract

Our objective was to describe weight, fat mass, skeletal mass and waist gains across the adult lifespan. We analysed data from 11 611 participants in the Brazilian Longitudinal Study of Adult Health, aged 35–74 years at baseline and followed from 2008 to 2024. Anthropometric measurements were performed at all visits, and bioimpedance at follow‐up visits. Weight at age 20 was self‐reported. Age‐related changes in weight, fat and muscle mass and waist circumference were estimated using generalised estimating equations with restricted cubic splines. Participants' weight increased a mean of 15 kg from age 20–60, when weight peaked. Most of this increase (50%) occurred before age 35. While obesity manifested mainly in later life, maximum annual weight gains were seen up to age 40–45. Gains then tapered, and weight stabilised across ages 55–65, and then decreased at older ages. While fat and muscle mass followed trajectories similar to that of weight, waist circumference increased steadily up to age 75. Approximately half of the difference in adult weight occurred before age 35. Population‐based and clinical interventions to limit weight gain in young adulthood, if effective, could have a major impact on the incidence and long‐term burden of obesity

## Study Importance

- What Is Already Known About This Subject?
  - While newer treatments for obesity can induce substantial weight loss, long‐term weight maintenance remains a major problem, underscoring the importance of preventing obesity. Most studies show weight gain in early adulthood, stabilisation at midlife and frequently loss thereafter. But evidence is scarce outside high‐income settings.
- What This Study Adds?
  - In a large Brazilian cohort, we comprehensively captured weight change across adulthood, finding substantial weight and fat mass gains beginning at age 20, concentrated in the first 15 years of adulthood. Weight stabilised or declined across ages 55–65, with bioimpedance measures of fat mass confirming these trends. To prevent obesity onward, greater attention in public health policies and trials should be given to preventing early adult weight gain.

## 1 Introduction

Excess adiposity is currently the sixth leading risk factor causing disease burden worldwide. Additionally, high blood pressure and hyperglycaemia—both of which have obesity as a major, potentially modifiable risk factor—rank as the second and fifth leading risk factors, respectively. Among major risk factors, obesity has had the most rapidly increasing burden, with an 81% increase in crude burden from 1990 to 2021 [1]. Approximately 878 million people worldwide are currently living with obesity, according to the NCD Risk Factor Collaboration [2].

While traditional approaches to weight loss have usually failed to produce the desired loss, newer treatments for managing overweight and obesity, such as more aggressive nutrition interventions with total diet replacement [3, 4] and GLP‐1 agonist therapy [5, 6] produce significant weight loss, improvement in metabolic parameters, and a reduced incidence of several diseases. However, in most settings, these approaches to obtaining large weight losses are not widely accessible, are infrequently applied, have not produced the same degree of weight loss in real‐world settings found in clinical trials, and introduce a great demand for health system resources. In addition, all weight loss treatments for obesity are usually limited by subsequent weight regain, resulting from mechanisms to defend the established weight [7, 8, 9].

Given these major weight loss and maintenance challenges, preventing weight gain or its early reversal, when mechanisms defending a state of excess weight may be less prominent, merits renewed attention. To document the trajectory of weight and fat mass gains throughout adult life, we leveraged data from the ELSA‐Brasil cohort, with its wide age range at baseline and multiple measurements over approximately 13 years of follow‐up. Our objective was to characterise weight gains across the adult lifespan and the extent to which it results from changes in fat or muscle mass.

## 2 Methods

### 2.1 Study Design and Participants

The Brazilian Longitudinal Study of Adult Health (ELSA‐Brasil) is a multicentre prospective occupational cohort comprising 15 105 adults, designed to investigate risk factors for chronic non‐communicable diseases, especially cardiovascular diseases and diabetes, over long‐term follow‐up. Participants were active or retired civil servants, aged 35–74 years, recruited from public universities and research institutions in six Brazilian state capitals (Salvador, Belo Horizonte, Rio de Janeiro, São Paulo, Vitória and Porto Alegre) [10]. The first visit occurred between 2008 and 2010, during which participants underwent a series of interviews and clinical and laboratory examinations.

We then invited participants to return for three follow‐up visits between 2012–2014, 2017–2019 and 2022–2024. Ethics committees from all institutions approved the research protocols, and participants provided written informed consent for all visits.

We excluded participants who did not attend the first two follow‐up visits, had missing data on any of the variables of interest, or reported bariatric surgery at baseline or at any time between visits. The final analytical sample consisted of 11 611 participants (Figure S1).

### 2.2 Measurements

We conducted participant interviews using standardised questionnaires to collect self‐reported information on age, sex, race/colour, educational attainment, family income, medical history, smoking status (current and past), alcohol consumption and physical activity [11]. Alcohol consumption was assessed using a standardised questionnaire developed for the ELSA‐Brasil study, which collected information on the weekly intake of beer, wine and spirits. Reported volumes were converted into grams of alcohol based on standard ethanol concentrations for each beverage type, and total alcohol intake was estimated by summing across all beverages. Physical activity was assessed using the long form International Physical Activity Questionnaire (IPAQ), considering leisure‐time and transport‐related domains [12].

After a 12‐h overnight fast, we performed anthropometric measurements following standardised protocols [11, 13]. Body weight (kg) was measured using an electronic scale with a maximum capacity of 200 kg (Toledo, São Bernardo do Campo, Brazil); waist circumference was assessed with a non‐elastic 150 cm tape (Mabis‐Gulick, Waukegan, IL, USA), positioned at the midpoint between the lowest rib margin and the iliac crest. In addition to the direct measurement of weight during visits, at baseline, we asked participants about their weight at the age of 20.

Body mass index (BMI) was calculated as weight (kg) divided by squared height (m^2^) and was used both as a continuous variable and as mutually exclusive categories: normal BMI (BMI ≥ 18.5 kg/m^2^), overweight (BMI ≥ 25 kg/m^2^), obesity (BMI ≥ 30 kg/m^2^) and obesity classes II and III (BMI ≥ 35 kg/m^2^) [14].

Body composition breakdown into fat and skeletal muscle mass was obtained at follow‐up visits using segmental bioimpedance with an 8‐electrode system (InBody 230; InBody Co., Seoul, Korea). We excluded individuals with pacemakers or metal prostheses from this measurement. Before measurement, participants were asked to urinate, wear standard clothing and remove all metallic objects. Trained professionals, certified before and throughout the study, performed measurements using pre‐established standard procedures, equipment and techniques. The exam agrees well with dual‐energy X‐ray absorptiometry, the gold standard for body composition assessment [15]. The sum of fat and skeletal muscle masses is less than total body weight, which also includes weight in organs, bones and other bodily fluids.

Measurements were conducted during all four visits, followed standardised protocols, and were subject to regular quality control assessments [11]. Bioimpedance was performed only during follow‐up visits.

### 2.3 Statistical Analysis

We described participant characteristics and outcomes overall and according to sex using absolute and relative frequencies for categorical variables and mean and standard deviation or median and 25th–75th percentiles for quantitative variables.

To describe the unadjusted distribution of BMI categories across adulthood, we calculated the frequencies of normal weight, overweight, obesity and class II obesity or higher at each age for participants at each visit. A stacked area plot was constructed, and frequencies were smoothed using smoothing splines (smooth.spline in R) to improve visualisation of age‐related patterns.

To describe age‐related changes in annual body weight, fat mass, skeletal muscle mass and waist circumference between ages 35 and 75 years, observations from the three between‐visit periods were stacked, allowing participants to contribute up to three repeated observations across different ages during follow‐up. For each period, annual change was calculated as the weight difference between consecutive visits divided by the corresponding follow‐up time in years.

To estimate annual weight gain from age 20–34, we subtracted self‐reported weight at age 20 from weight at age 35, with the difference divided by 15. As few participants entered the study at age 35, we estimated weight at age 35 for most by subtracting their estimated cumulative weight gain between ages 35 and their visit 1 age starting with their visit 1 baseline weight. To obtain this estimated cumulative gain for each participant, we summed the average annual weight gains of participants with data for the years from age 35 to the participant's age at baseline. These annual gains were calculated separately in each sex and baseline BMI strata. For example, as the estimated mean weight gain for normoweight women with data between years 35 and 40 was 2.2 kg, the estimated weight at age 35 for a woman 40 years old and weighing 65 kg at baseline was 62.8 kg (65–2.2 kg).

Adjusted age‐related changes were then estimated from these calculations using generalised estimating equations (GEE) with restricted cubic splines for age, accounting for within‐participant repeated measurements. Models were adjusted for sex, race/skin colour, educational level, income, BMI and data collection period. An autoregressive first‐order [AR(1)] working correlation structure was specified to account for the correlation between repeated observations over time. Predicted values and 95% confidence intervals (95% CI) derived from these models were used to generate the figures. The same modelling strategy was applied to estimate cumulative changes in body weight, fat mass, skeletal muscle mass and waist circumference across adulthood. We also calculated the percentage of total weight difference accumulated across adulthood, defined as the difference between mean self‐reported weight at age 20 and mean weight at age 60, when participants, on average, reached their maximum weight.

To explore potential birth cohort effects, participants were grouped into 10‐year birth cohorts, estimated from age and year of baseline assessment. The body weight trajectory was then calculated across age separately for each birth cohort and displayed graphically.

Statistical analyses were performed using RStudio (version 2026.04.0 + 526) and SAS OnDemand for Academics. In figures, data presented as age 75 represent all of the relatively few individuals aged 75 years or older.

## 3 Results

Table 1 (*n* = 11 611) presents the sample's baseline characteristics according to sex. Most participants were aged 40–59 years (72.6%), female (55.3%), self‐identified as white (52.7%), had a college/university education (54.9%) and reported never smoking (58.9%). Women, compared with men, showed a higher prevalence of insufficient physical activity levels (< 600 MET‐min/week; 69.6% vs. 58.6%), elevated waist circumference (70.3% vs. 51.4%) and obesity (22.9% vs. 19.4%). Anthropometric characteristics by age group are presented in Table S1. Baseline characteristics of participants excluded from the analytical sample, are presented in Table S2.

**TABLE 1:** Baseline demographic and clinical characteristics (*n* = 11 611).

Characteristics	Total (*n* = 11 611)	Men (*n* = 5189, 44.7%)	Women (*n* = 6422, 55.3%)
Age groups (years)			
34–39	935 (8.1%)	456 (8.8%)	479 (7.5%)
40–49	4320 (37.2%)	1980 (38.2%)	2340 (36.4%)
50–59	4107 (35.4%)	1749 (33.7%)	2358 (36.7%)
60–69	1882 (16.2%)	796 (15.3%)	1086 (16.9%)
70–74	365 (3.1%)	207 (4.0%)	158 (2.5%)
Race/skin colour			
Black	1854 (16.0%)	709 (13.7%)	1145 (17.8%)
Brown	3222 (27.7%)	1541 (29.7%)	1681 (26.2%)
White	6120 (52.7%)	2772 (53.4%)	3348 (52.1%)
Asian	301 (2.6%)	101 (1.9%)	200 (3.1%)
Indigenous	114 (1.0%)	66 (1.3%)	48 (0.7%)
Educational level			
Less than elementary	530 (4.6%)	321 (6.2%)	209 (3.3%)
Elementary	707 (6.1%)	402 (7.7%)	305 (4.7%)
Secondary	3994 (34.4%)	1704 (32.8%)	2290 (35.7%)
College/university	6380 (54.9%)	2762 (53.2%)	3618 (56.3%)
Income (*reais*)	1411 (726–2282)	1349 (726–2075)	1452 (747–2282)
Smoking			
Never	6836 (58.9%)	2757 (53.1%)	4079 (63.5%)
Former smoker	3394 (29.2%)	1786 (34.4%)	1608 (25.0%)
Current smoker	1381 (11.9%)	646 (12.4%)	735 (11.4%)
Physical activity			
≥ 600 MET‐min/week	4097 (35.3%)	2146 (41.4%)	1951 (30.4%)
< 600 MET‐min/week	7514 (64.7%)	3043 (58.6%)	4471 (69.6%)
Alcohol consumption (g/day)	0 (0–67.2)	39 (0–119)	0 (0–30)
Weight (kg)	73.2 (14.7)	79.8 (14.1)	67.8 (12.9)
Waist circumference			
≥ 94 cm for men, ≥ 80 cm for women	7181 (61.8%)	2669 (51.4%)	4512 (70.3%)
< 94 cm for men, < 80 cm for women	4430 (38.2%)	2520 (48.6%)	1910 (29.7%)
BMI category (kg/m^2^)			
Normal BMI	4428 (38.1%)	1809 (34.9%)	2619 (40.8%)
Overweight	4707 (40.5%)	2374 (45.8%)	2333 (36.3%)
Obesity class I	1852 (16.0%)	806 (15.5%)	1046 (16.3%)
Obesity class II or higher	624 (5.4%)	200 (3.9%)	424 (6.6%)

*Note:* Categorical variables are expressed as the absolute number (percentage). Weight is expressed as mean (standard deviation). Income and alcohol are expressed as median (quartile 1‐quartile 3).

**FIGURE 1:**
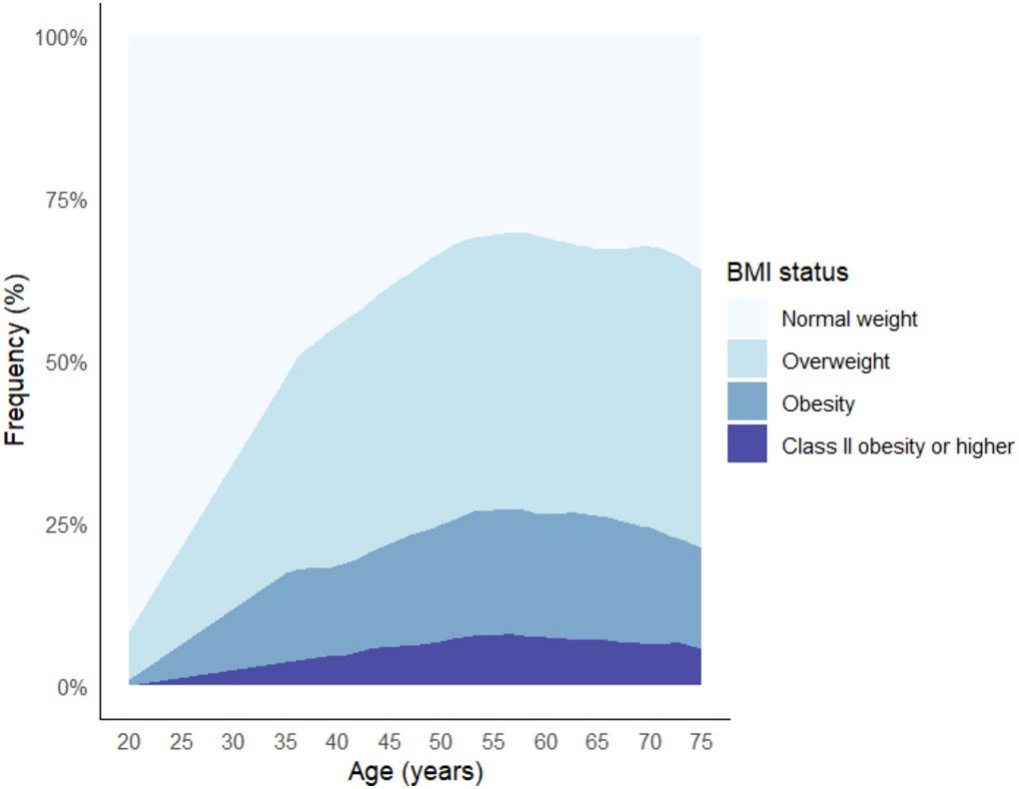
Frequency of BMI status over the adult lifespan. ELSA‐Brasil, 2008–2024.

Figure 1 illustrates the distribution of BMI categories (normal BMI, overweight, obesity and class II obesity or higher) across different ages. In the first decade of adult life, most individuals had a normal weight. However, as age increased, the prevalence of overweight and obesity rose sharply and peaked between ages 50–60, at which point these categories encompassed approximately two‐thirds of the sample. Their prevalence then declined slightly. The proportion of class II or higher obesity remained relatively low across all ages and followed a pattern similar to overall obesity.

**FIGURE 2:**
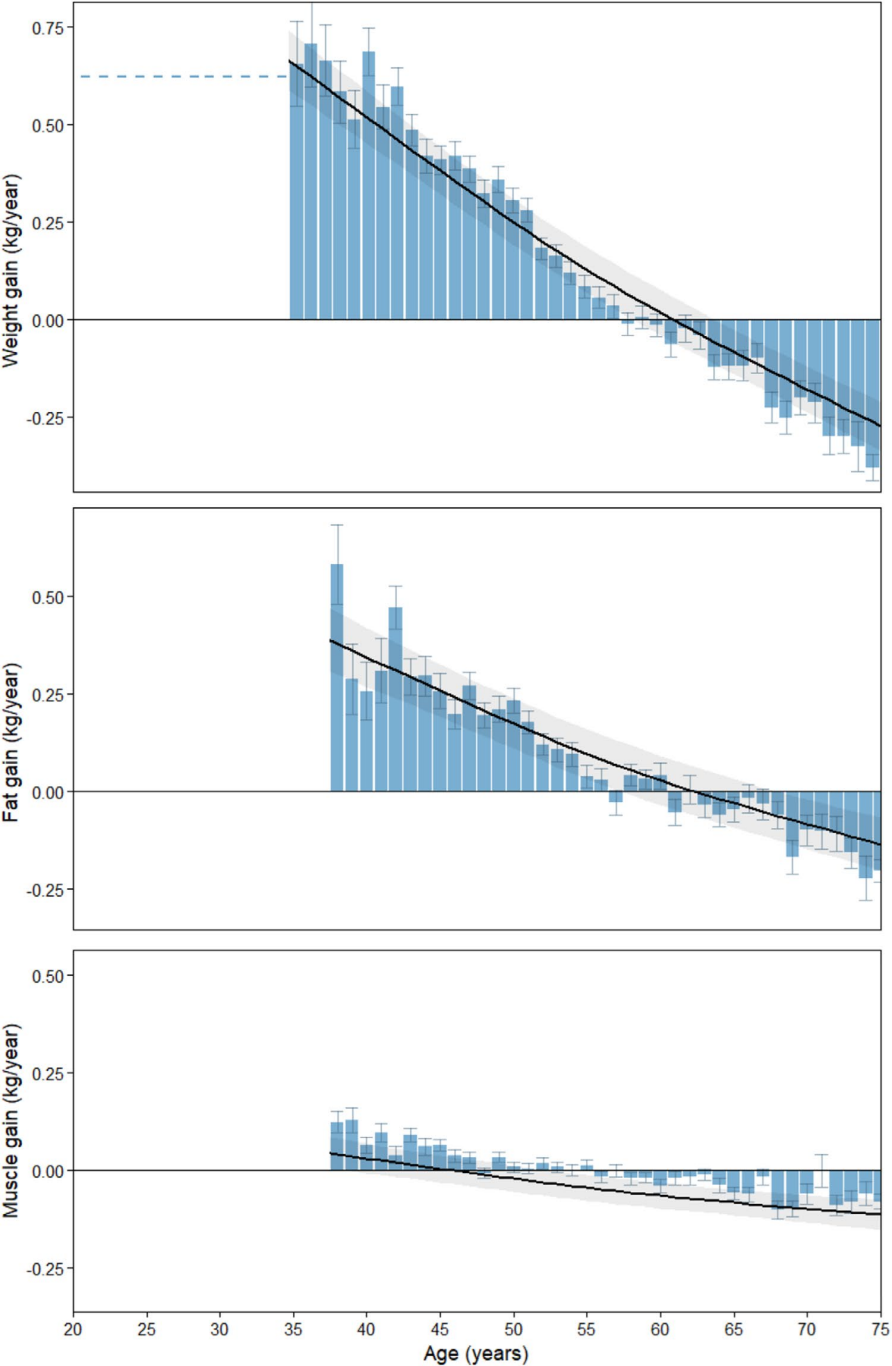
Annual weight, fat and muscle gain (kg/year) by age (years) across adult life. The upper, middle and lower panels represent weight gain, fat gain and muscle gain, respectively. Bars represent the observed mean annual gain for each age, with error bars indicating 95% confidence intervals (95% CI). The solid line represents the adjusted restricted cubic spline estimated using generalised estimating equations (GEE), and the shaded area represents the corresponding 95% CI. Models were adjusted for sex, race/skin colour, education, income, BMI and data collection period. The dashed line represents the estimated mean annual gain between ages 20 and 34 years based on retrospectively reported weight at 20 years of age. ELSA‐Brasil, 2008–2024.

Figure 2 presents average weight (kg/year) changes over the adult age span and underlying changes in fat and muscle mass from the youngest age at Visit 2 for all 11 611 participants. Weight increased annually from age 20 until age 57, followed by a subsequent decline. The annual weight gain up to 40 was notably greater than in later years. After the age of 57–58, weight, on average, decreased, with weight losses being more pronounced with advancing age. The adjusted annual weight gain from ages 20 to 34 was 0.50 kg/yr (95% CI 0.45–0.55 kg/yr). Gains continued at this level to age 57, after which they declined progressively (Table S3). Adjustment attenuated the weight changes at extreme ages.

When testing an interaction between age at measurements and birth cohort group, we observed an inverse association, with younger cohorts showing larger weight gains (*p* < 0.01 for the 35–44 and 45–54‐year cohorts compared with the 65–74‐year cohort).

Fat and muscle gain trajectories followed a similar pattern, although data were available only from ages 37–38 onward. Once again, gains were highest at the youngest ages and steadily declined afterwards. Muscle mass stabilised from the late 40s to the late 50s and slowly decreased thereafter. Gains in fat mass continued into the late 50s, with a steady subsequent decline. Gains and losses in fat mass were notably greater than those of skeletal muscle mass (Figure 2). Figure S2 displays the same data now stratified by sex (men = 5189; women = 6422). The patterns are similar to those observed above.

Waist circumference gains were also highest in the late 30s and early 40s. However, unlike the above measures, average waist circumference increased to 75, the highest age recorded (Figure S3).

**FIGURE 3:**
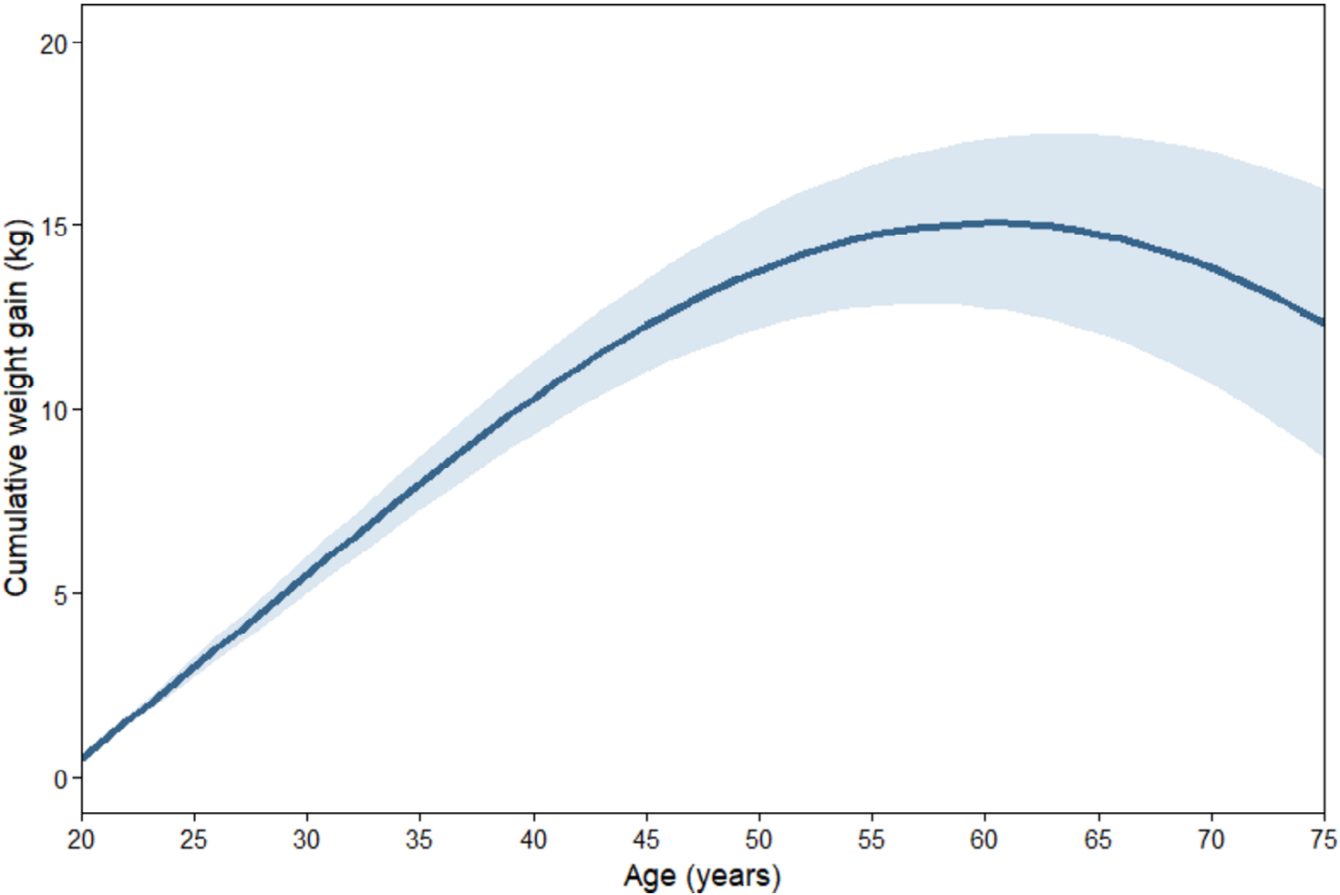
Estimated cumulative weight gain (kg) across ages 20–75 years based on the adjusted restricted cubic spline model. The curve was estimated using generalised estimating equations (GEE) and adjusted for sex, race/skin colour, education, income, BMI and data collection period. ELSA‐Brasil, 2008–2024.

Figure 3 shows the adjusted average cumulative difference in weight from ages 20 to 75. In consonance with the pattern of annual weight gains, this difference increased steadily until approximately age 40, then slowed slightly, reaching a maximum of 14–15 kg at ages 52–60 and then gradually declining to 12 kg at age 75. As seen in Figure S4, of this total difference, 7.5 kg (50%) occurred between the ages of 20 and 34, 4.4 kg (29%) during ages 35–44 and 3.1 kg (21%) from age 45 to 60.

**FIGURE 4:**
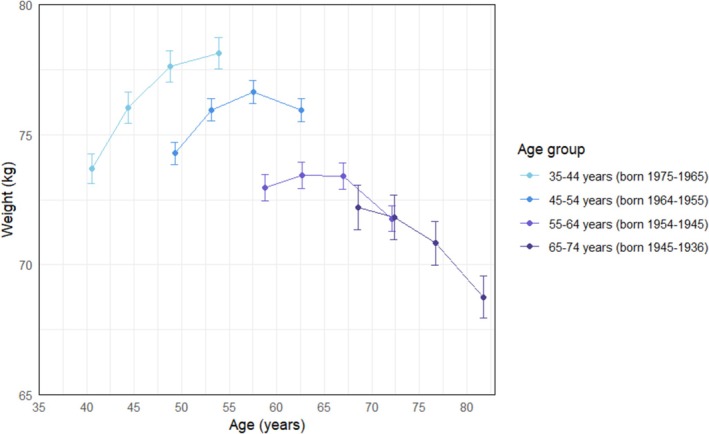
Mean weight (kg) according to age across birth cohorts. Points represent observed means and error bars indicate 95% confidence intervals. ELSA‐Brasil, 2008–2024.

Figure 4 presents weight gain according to birth cohort groups at study entry. Relative gains of different cohorts in overlapping age segments document greater weight gains in younger birth cohorts, consistent with the interaction found above. All birth cohorts with data beyond age 60 showed declines at older ages.

The adjusted cumulative differences in fat mass from age 38 (the youngest age at Visit 2) peaked at 5.1 kg at age 64 (Figure S5), representing approximately 93% of weight difference from age 38 to peak, and that of skeletal muscle mass peaked at 0.21 kg at age 49 (Figure S6). In contrast, the difference in waist circumference continued to increase throughout the age span, reaching 24.9 cm at age 75 (Figure S7). Adjustment here increased the average gain at older ages.

## 4 Discussion

In this 13‐year follow‐up of Brazilian adults, we found an estimated 15 kg increase in weight across the adult lifespan, assessed from 20 years to the cohort's age of peak weight. Yearly gains were greatest up to approximately age 40, then progressively decreased to the early fifties. Over the next decade, the average weight remained stable, then declined slightly from the mid‐sixties onward. In contrast, central obesity increased throughout the age span studied. Annual gains in fat mass paralleled weight changes; those in skeletal muscle mass, though smaller, were similarly directed. The larger weight gains in more recent birth cohorts, seen when the age range of cohorts overlapped, and the attenuation of changes when adjusted for calendar year of measurement demonstrate that, if current trends continue, the cumulative weight gain across adult life will most likely be considerably higher for the more recent birth cohorts.

Our findings are consistent with other studies of adult weight change, indicating a large increase in body weight from young adulthood to midlife, followed by tapering and finally declining at older ages. A study involving more than 2 million English individuals aged 18–74 followed from 1998 to 2016 via a clinical practise database showed the largest gains in the youngest adults. Men with normal baseline weight gained a median of 0.79 kg/yr from age 18 to 24, then 0.45 kg/yr when aged 25–34. Gains then decreased slightly to 0.4 kg/yr between ages 35 and 44 and markedly to only 0.05 kg/yr between ages 65 and 74. Women showed a similar pattern, though with lesser gains and a slight annual loss at ages 65–74. Gains were slightly lower in individuals with overweight and notably less in those with obesity at baseline, with these groups losing weight in the 65–74 age strata [16]. Similarly, a composite analysis of German cohorts with baseline exams from 1994 to 2007 and 4–12 year follow‐ups found the largest weight gains in young adults and weight losses from age 65 onward [17]. A Swedish cohort of women initially aged 29–49, followed from 1991 to 2003 through self‐reported weights, showed slightly greater gains among younger women [18]. Another study from northern Norway covering the period of 2003–2014, with 3496 participants aged 36 to 69 years and including a high proportion of Indigenous individuals, reported relatively large (~0.3 kg/yr) annual weight gains in those aged 36–44 at entry, which then rapidly decreased to minimal gains at ages 55–59 and progressively increasing losses thereafter [19]. In the USA, data from NHANES collected from 2013 to 2018 showed that weight gain decreased from 0.69 kg/yr in the early to midlife adult years to 0.14 kg/yr in midlife to late adulthood [20].

Notably, the above findings come from high‐income countries. Widespread obesity, however, is also now a frequent phenomenon in low‐ and middle‐income countries (LMICs), where a compressed nutritional transition has led over recent decades to an explosion of excess weight. Yet, data on weight gain in adult life from such countries are scarce and not completely consistent. In Malaysia, nationally representative respondents (≥ 18 years) were pooled from four cross‐sectional surveys conducted between 1996 and 2015 to construct a picture of BMI change over age in 94 537 adults [21]. Adjusted for cohort effects, BMI trajectories increased steeply from age 18–65 years (from 18 kg/m^2^ to approximately 25 kg/m^2^ for men and 27 kg/m^2^ for women), with subsequent plateaus [21]. A large Chinese cohort, followed from 2006 to 2019, also found similar findings. Though not presenting weight changes directly, the study documented a progressively decreased risk of reaching greater BMI categories from ages 18–24 to 65–74 years [22]. In contrast, a representative longitudinal study conducted in Tehran from 1999 to 2015 with 4895 participants aged ≥ 20 years followed over a 15‐year follow‐up period differed somewhat, with much smaller overall weight differences (men: 5.3 kg, women: 4.1 kg) across ages, with no decline at older ages [23].

Our findings span a more recent period (2008–2024) and thus provide more current information on the trends and their implications for the continuing global rise in obesity. Early weight gain has been shown to produce serious repercussions. Even when not resulting in obesity in early adulthood, the weight gained, by elevating a healthy BMI into the overweight range, permits many individuals to reach a BMI classified as obesity with only small additional weight gain in later life [16]. In addition, a follow‐up of more than 36 000 NHANES participants found that weight gain from young to middle adulthood, followed by a stable obesity in later life, was associated with an increased risk of all‐cause mortality and heart disease mortality; in contrast, obesity attained later in life was not associated with increased mortality [24]. Similarly, a study of Chinese subjects showed that the risk of developing diabetes was higher with greater weight gain early on.

Given these findings, obesity must be recognised as a life course disease, with its antecedents requiring public health attention long before obesity onset. Establishing healthy eating patterns and adequate physical activity through population‐based interventions remains underemphasised and underfunded. Actions to increase access to foods that are not ultra‐processed, for example through their inclusion in school lunch programs [25], as well as taxes and subsidies to make healthy foods more accessible and unhealthy ones less so [26] are needed. Evidence shows that certain dietary patterns are associated with weight gain and adverse metabolic outcomes, while others are associated with more favourable trajectories [27, 28], and public health strategies should support healthier choices at the population level.

Public health messaging in early adulthood should emphasise health and well‐being, including awareness of weight changes over time [29], while prioritising supportive, non‐stigmatising approaches. Interventions should focus on promoting sustainable behaviours and healthy environments, rather than narrowly targeting weight outcomes. It is also important to acknowledge that weight regulation is influenced by biological and other factors beyond individual control, which should be considered in the design of prevention strategies. In all actions, avoiding stigma and an excessive focus on weight, body shape and food must remain a central principle [30].

Within the clinical context, treatment of obesity, the primary focus of weight control to date, has been hampered in the long run by feedback mechanisms producing decreased energy expenditure and increased appetite to defend an established weight [7, 8, 9]. A qualitative study has highlighted a fact well known to clinicians—that the burden of working to maintain weight once lost is considerably greater than maintaining a healthy weight [29] Although how long a period after a weight gain is required for these defences to activate remains unknown, the avoidance of small weight gains and actions to produce their rapid remission in early adult life could be a major alternative focus for confronting the obesity epidemic.

Clinical trials to date on the prevention of weight gain in individuals without obesity are limited. While they document the benefit of intervention, reductions in gains are small and long‐term follow‐up is usually lacking [31]. Our findings, together with what was previously known about these gains and their prevention, suggest that trials of more frequent monitoring of weight gain in early to mid‐adulthood, with lifestyle and pharmacologic interventions to eliminate short‐term gains when detected, accompanied by counselling on how to avoid future weight gain, should be stimulated. The advent of more effective options for weight management and control [5, 6], assuming their increasing accessibility, offers new opportunities for such approaches.

Our study has limitations. First, unlike the weight we measured from age 35 onward, weight at age 20 was self‐reported and therefore subject to recall and social desirability biases. These biases may not operate uniformly across participants, as older individuals are required to recall weight over a longer period, which may increase measurement error. In addition, changes in societal norms and perceptions regarding body weight across birth cohorts may differentially influence reporting accuracy. Additionally, period and cohort effects do not permit the assertion that our findings at older ages are likely to be what will be experienced by younger cohort members as they age. As a result, estimates of weight change over the life course may be affected by differential misclassification, potentially influencing comparisons across age groups and cohorts. However, previous studies have suggested that self‐reported past weight, particularly in early adulthood, tends to be reasonably valid at the population level [32]. Second, our study is a long‐term cohort naturally subject to attrition, potentially adding bias. However, the loss to follow‐up was only 18%, considerably less than most studies cited above. It is worth noting that individuals lost to follow‐up had a higher prevalence of obesity and characteristics (e.g., lower education and income levels) likely related to weight gain. Consequently, our findings may underestimate the cohort's weight gain and perhaps also greater early weight gain. Third, we did not investigate diseases or conditions that may influence weight change across adulthood, such as mood and eating disorders. Fourth, though our weight loss data from age 35 onwards was contemporary, no participant's weight change covered the whole adult lifespan. Thus, similar to all reported cohort studies on this issue, our cumulative findings, while representative of our ELSA‐Brasil cohort as a whole, are best described as cumulative differences in weight and not cumulative gains across the adult age span. While greater gains in more recent birth cohorts suggest that the problem of weight gain among young adults is increasing, they exaggerate somewhat the percentage of adult weight gain we estimated to have occurred in early adult life. Fifth, the ELSA‐Brasil cohort is composed of active and retired civil servants, who generally have higher educational attainment and more stable socioeconomic conditions than the overall Brazilian population. This may limit the generalisability of our findings, as patterns of weight gain and associated factors may differ across different socio‐economic segments of the population.

Several strengths of our study need highlighting. To our knowledge, ours is the only contemporary cohort in a LMI country to characterise weight change across the total adult age range. Anthropometric measurements followed standardised protocols and were conducted by trained staff rather than being obtained by self‐report, thus enhancing their accuracy. Moreover, we also documented consistent fat and muscle mass changes underlying the weight changes in addition to the studies describing weight change across the lifespan. Additionally, our study included participants across a wide age range, from 35 to 74 years and a 13‐year follow‐up period. This allowed us, considering the self‐report of weight at age 20, to comprehensively capture the cohort's weight trajectory across the lifespan, providing a more complete picture of long‐term weight and body composition changes through four weight measurements and two bioimpedance measurements per participant.

In conclusion, our study, like those of high‐income countries and most in LMICs, showed that untoward gains in the body weight of contemporary Brazilians adults occurred principally in early on, with lesser gains in midlife to the late 50s, after which decline began. The patterns of fat and muscle mass accumulation exhibited a similar trajectory to that of body weight, while central obesity continued to increase to the maximum age of our ascertainment. Although obesity was more prevalent among older participants, weight gain was most notable in younger ones, setting the stage for obesity in later life. Finally, our findings underline the opportunity that exists for implementing measures to prevent or rapidly reverse gains in early adulthood. Greater attention should be given to public health interventions stimulating weight maintenance through healthy eating and adequate physical activity. Additionally, public health messaging should focus on avoiding these smaller but frequent gains in early adult life. Clinical trials should be implemented to test the feasibility of short‐term weight loss treatment combined with lifestyle counselling to maintain a healthy weight across early adult life.

## Author Contributions

Conceptualisation: B.B.D. and M.I.S. Methodology: B.B.D. and M.I.S. Formal analysis: S.L.C. Investigation: S.L.C. Data curation: S.L.C., P.A.B. and B.B.D. Writing – original draft preparation: S.L.C., B.B.D. and M.I.S. Writing – review and editing: P.A.B., M.J.F. and M.D.C.B.M. Project administration: B.B.D., M.I.S. and M.J.F. Funding acquisition: B.B.D., M.I.S., M.D.C.B.M. and M.J.F. S.L.C. has directly accessed and verified the underlying data reported in the manuscript. All authors have read and agreed to the published version of the manuscript.

## Funding

This research received support from the Brazilian Ministries of Health (Department of Science and Technology) and of Science, Technology and Innovation (Funding Authority for Studies and Projects—FINEP, grant numbers 01 06 0010.00, 01 06 0212.00, 01 06 0300.00, 01 06 0278.00, 01 06 0115.00, 01 06 0071.00) and the National Council for Scientific and Technological Development (CNPq, grant numbers 405551/2015‐0, 405544/2015‐4, 405547/2015‐3, 405552/2015‐7, 405543/2015‐8, 405545/2015‐0). S.L.C. received a fellowship from the Brazilian National Council for Scientific and Technological Development (CNPq, grant number 150805/2024‐1). M.J.F., M.D.C.B,M., M.I.S. and B.B.D. received productivity in research fellowships from CNPq. Researchers were independent of the funders. The study sponsors had no role in the collection, analysis or interpretation of data; in the writing of the report; or in the decision to submit the paper for publication.

## Ethics Statement

The study was conducted in accordance with the Declaration of Helsinki. Ethics committees of each institution approved the research protocol, and subjects gave written consent to participate in each visit. The study approved by the Research Ethics Committee of ISC‐UFBA (ELSA‐Brasil Bahia) CAAE number 0017.1.069.000‐06 Approval Date 26 May 2006; Research Ethics Committee of FIOCRUZ (ELSA‐Brasil Rio de Janeiro) CAAE number 0058.0.011.000‐07 Approval Date 18 September 2006; Research Ethics Committee of Hospital Universitário‐USP (ELSA‐Brasil São Paulo) CAAE number 0016.1.198.000‐06 Approval Date 19 May 2006; Research Ethics Committee of UFMG (ELSA‐Brasil Minas Gerais) CAAE number 0186.1.203.000‐06 Approval Date 21 June 2006; Research Ethics Committee of Centro de Ciências de Saúde da UFES (ELSA‐Brasil Espírito Santo) CAAE number 08109612.7.2003.5060 Approval Date 31 May 2006; Research Ethics Committee of Hospital de Clínicas de Porto Alegre (ELSA‐Brasil Rio Grande do Sul) CAAE number 0017.1.069.000‐06 Approval Date 15 May 2006.

## Consent

Informed consent was obtained from all subjects involved in the study.

## Conflicts of Interest

The authors declare no conflicts of interest.

## Supporting information


**Table S1:** Baseline anthropometric measures and clinical conditions by age group, ELSA‐Brazil, 2008–2010 (*n* = 11 611).
**Table S2:** Baseline demographic and clinical characteristics of participants excluded from the analytical sample (*n* = 3494).
**Table S3:** Adjusted annual gains in weight, waist, fat and muscle mass by age group.
**Figure S1:** Participant flow diagram.
**Figure S2:** Annual weight, fat and muscle gain (kg/year) by age (years), stratified by sex. The upper, middle and lower panels represent weight gain, fat gain and muscle gain, respectively. Bars show the observed mean annual gain for each age, while the solid line represents the adjusted restricted cubic spline estimated using generalised estimating equations (GEE). Models were adjusted for race/skin colour, education, income, BMI and data collection period. Left: men; Right: women.
**Figure S3:** Annual waist gain (cm/year) across age. Bars show the observed mean annual waist gain for each age, while the solid line represents the adjusted restricted cubic spline estimated using generalised estimating equations (GEE). Models were adjusted for sex, race/skin colour, education, income, BMI and data collection period.
**Figure S4:** Percentage of weight gain ascertained at different ages across the adult lifespan. ELSA‐Brasil, 2008–2024.
**Figure S5:** Estimated cumulative fat mass gain (kg) across ages 35–75 years based on the adjusted restricted cubic spline model.
**Figure S6:** Estimated cumulative muscle mass gain (kg) across ages 35–75 years based on the adjusted restricted cubic spline model.
**Figure S7:** Estimated cumulative waist circumference gain (cm) across ages 35–75 years based on the adjusted restricted cubic spline model. The curve was estimated using generalised estimating equations (GEE) and adjusted for sex, race/skin colour, education, income, BMI and data collection period. ELSA‐Brasil, 2008–2024.

## Data Availability

Considering guidelines placed by the ethics committees responsible for ELSA's study centres, the deidentified participant data used in this study can be made available by request to ELSA's Publications Committee (gfeiden@gmail.com). Additional information can be obtained from the ELSA Co‐ordinator from the Research Center of Rio Grande do Sul (maria.schmidt@ufrgs.br). Study manuals and data dictionaries are available online (http://elsabrasil.org/pesquisadores/) and study protocols have been published (https://doi.org/10.1093/aje/kwr294 and https://doi.org/10.1093/ije/dyu027).
