# Supplementary material for: Weight Gains Across Adulthood—The ELSA‐Brasil Study
Source: Clin Obes. 2026 Jul 7;16(4):e70095. doi: 10.1111/cob.70095 (PMC13341270; doi:10.1111/cob.70095)
Supplement: Supplementary file 1 — Table S1: Baseline anthropometric measures and clinical conditions by age group, ELSA‐Brazil, 2008–2010 (n = 11 611). Table S2: Baseline demographic and clinical characteristics of participants excluded from the analytical sample (n = 3494). Table S3: Adjusted annual gains in weight, waist, fat and muscle mass by age group. Figure S1: Participant flow diagram. Figure S2: Annual weight, fat and muscle gain (kg/year) by age (years), stratified by sex. The upper, middle and lower panels represent weight gain, fat gain and muscle gain, respectively. Bars show the observed mean annual gain for each age, while the solid line represents the adjusted restricted cubic spline estimated using generalised estimating equations (GEE). Models were adjusted for race/skin colour, education, income, BMI and data collection period. Left: men; Right: women. Figure S3: Annual waist gain (cm/year) across age. Bars show the observed mean annual waist gain for each age, while the solid line represents the adjusted restricted cubic spline estimated using generalised estimating equations (GEE). Models were adjusted for sex, race/skin colour, education, income, BMI and data collection period. Figure S4: Percentage of weight gain ascertained at different ages across the adult lifespan. ELSA‐Brasil, 2008–2024. Figure S5: Estimated cumulative fat mass gain (kg) across ages 35–75 years based on the adjusted restricted cubic spline model. Figure S6: Estimated cumulative muscle mass gain (kg) across ages 35–75 years based on the adjusted restricted cubic spline model. Figure S7: Estimated cumulative waist circumference gain (cm) across ages 35–75 years based on the adjusted restricted cubic spline model. The curve was estimated using generalised estimating equations (GEE) and adjusted for sex, race/skin colour, education, income, BMI and data collection period. ELSA‐Brasil, 2008–2024. [file COB-16-e70095-s001.pdf]

SUPPLEMENTARY FILE

Weight gains across adulthood – the ELSA-Brasil study

Scheine L Canhada PhD, Paula A Bracco PhD, Maria de Jesus Fonseca PhD, Maria Del Carmen Bisi Molina PhD, Maria Inês Schmidt MD, Bruce B Duncan MD

Contact information: scheinelc@gmail.com

Supplementary Table S1. Baseline anthropometric measures and clinical conditions by age group, ELSA-Brazil, 2008-2010 (n=11611).

	Age Group				
**Characteristics**	**35-44 years**	**45-54 years**	**55-64 years**	**65-74 years**	**Total**
	n=2751	n=4727	n=3103	n=1030	n=11611
**BMI category (kg/m^2)**					
Normal BMI	1251 (45.5%)	1743 (36.9%)	1085 (35.0%)	349 (33.9%)	4428 (38.1%)
Overweight	1019 (37.0%)	1931 (40.9%)	1312 (42.2%)	445 (43.2%)	4707 (40.6%)
Obesity	481 (17.5%)	1053 (22.3%)	706 (22.8%)	236 (22.9%)	2476 (21.3%)
**Weight (kg)**	73.7 (15.4)	74.3 (14.8)	73.0 (14.2)	72.2 (13.9)	73.6 (14.7)
**Waist (cm)**	87.4 (12.3)	90.6 (12.3)	92.2 (12.4)	94.0 (12.4)	90.6 (12.5)

Weight and waist circumference are expressed as mean (standard deviation). Categorical variables are expressed as absolute number (percentage).

Supplementary Table S2. Baseline demographic and clinical characteristics of participants excluded from the analytical sample (n = 3494)

Characteristics	Analytical sample (n=11611)	Excluded sample (n=3494)	P-value
**Age groups (years)**			<0.001
34-39	935 (8.1%)	221 (6.3%)	
40-49	4320 (37.2%)	978 (28.0%)	
50-59	4107 (35.4%)	1281 (36.7%)	
60-69	1882 (16.2%)	768 (22.0%)	
70-74	365 (3.1%)	246 (7.0%)	
**Sex**			<0.001
Women	6422 (55.3%)	1796 (51.4%)	
**Race/skin color**			0.042
Black	1854 (16.0%)	543 (16.4%)	
Brown	3222 (27.7%)	980 (29.6%)	
White	6120 (52.7%)	1671 (50.5%)	
Asian	301 (2.6%)	73 (2.2%)	
Indigenous	114 (1.0%)	43 (1.3%)	
**Educational level**			<0.001
Less than elementary	530 (4.6%)	364 (10.4%)	
Elementary	707 (6.1%)	321 (9.2%)	
Secondary	3994 (34.4%)	1239 (35.5%)	
College/university	6380 (54.9%)	1570 (44.9%)	
**Income (*reais*)**	1411 (726- 2282)	1245 (692-2282)	0.001
**Smoking**			<0.001
Never	6836 (58.9%)	1758 (50.3%)	
Former smoker	3394 (29.2%)	1139 (32.6%)	
Current smoker	1381 (11.9%)	596 (17.1%)	
**Physical activity**			<0.001
⩾ 600 MET-min/week	4097 (35.3%)	1035 (31.6%)	
< 600 MET-min/week	7514 (64.7%)	2242 (68.4%)	
**Alcohol consumption (g/day)**	0 (0-67)	0 (0-72)	0.873
**Weight (kg)**	73.2 (14.7)	75.4 (16.3)	<0.001
**Waist circumference**			<0.001

⩾ 94cm (men), ⩾ 80cm (women)	7181 (61.8%)	2318 (66.4%)	
< 94cm (men), < 80cm (women)	4430 (38.2%)	1173 (33.6%)	
**BMI category (kg/m²)**			<0.001
Normal BMI	4428 (38.1%)	1138 (32.6%)	
Overweight	4707 (40.5%)	1371 (39.3%)	
Obesity class I	1852 (16.0%)	665 (19.1%)	
Obesity class II or higher	624 (5.4%)	314 (9.0%)	

Categorical variables are expressed as the absolute number (percentage). Weight is expressed as mean (standard deviation). Income and alcohol are expressed as median (quartile 1-quartile 3). P-values were calculated using chi-squared, Student's t, or Wilcoxon rank-sum tests, as appropriate.

Supplementary Table S3. Adjusted annual gains in weight, waist, fat and muscle mass by age group.

Age group	Weight (kg)	Fat (kg)	Muscle (kg)	Waist (cm)
20-34 years	0.50 (0.45-0.55)	---	---	---
35-39 years	0.47 (0.42-0.52)	0.26 (0.20-0.33)	0.02 (-0.02-0.05)	0.79 (0.72-0.86)
40-44 years	0.41 (0.36-0.47)	0.26 (0.19-0.33)	0.02 (-0.02-0.05)	0.79 (0.72-0.86)
45-49 years	0.32 (0.26-0.38)	0.25 (0.19-0.32)	0.01 (-0.02-0.05)	0.77 (0.70-0.84)
50-54 years	0.21 (0.15-0.28)	0.17 (0.11-0.23)	-0.01 (-0.05- 0.02)	0.68 (0.61-0.75)
55-59 years	0.09 (0.02-0.16)	0.05 (-0.01-0.11)	-0.04 (-0.07- -0.01)	0.58 (0.51-0.64)
60-64 years	-0.03 (-0.11-0.04)	0.02 (-0.05-0.08)	-0.05 (-0.09- -0.02)	0.51 (0.44-0.57)
65-69 years	-0.16 (-0.25- -0.07)	-0.03 (-0.09-0.04)	-0.07 (-0.11- -0.04)	0.44 (0.37- 0.51)
70-74 years	-0.28 (-0.38- -0.18)	-0.09 (-0.16- -0.03)	-0.09 (-0.13- -0.06)	0.37 (0.30-0.44)

Measures are presented as adjusted means (95% confidence interval). Adjusted for age, sex, race/skin color, education, income, BMI, and data collection period.

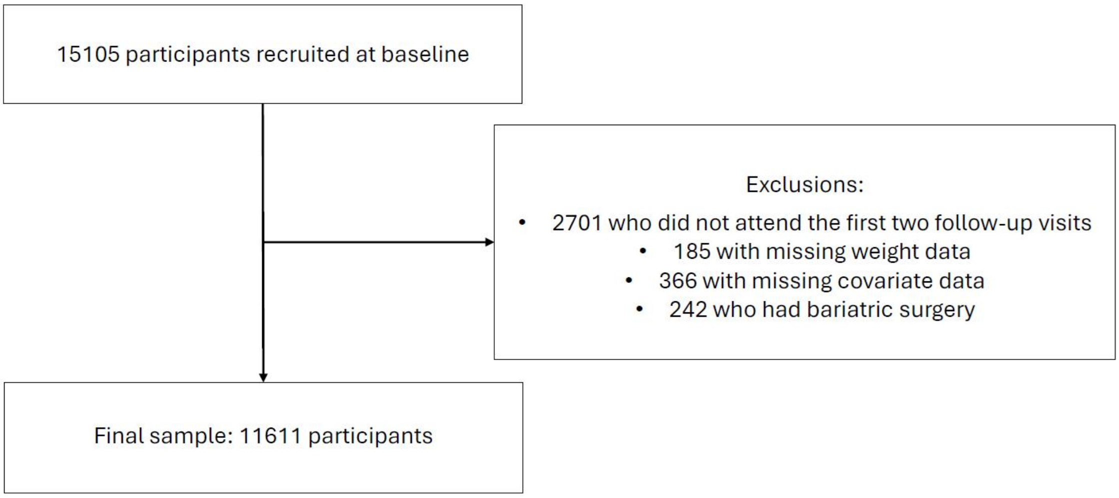

Supplementary Figure S1. Participant Flow Diagram.

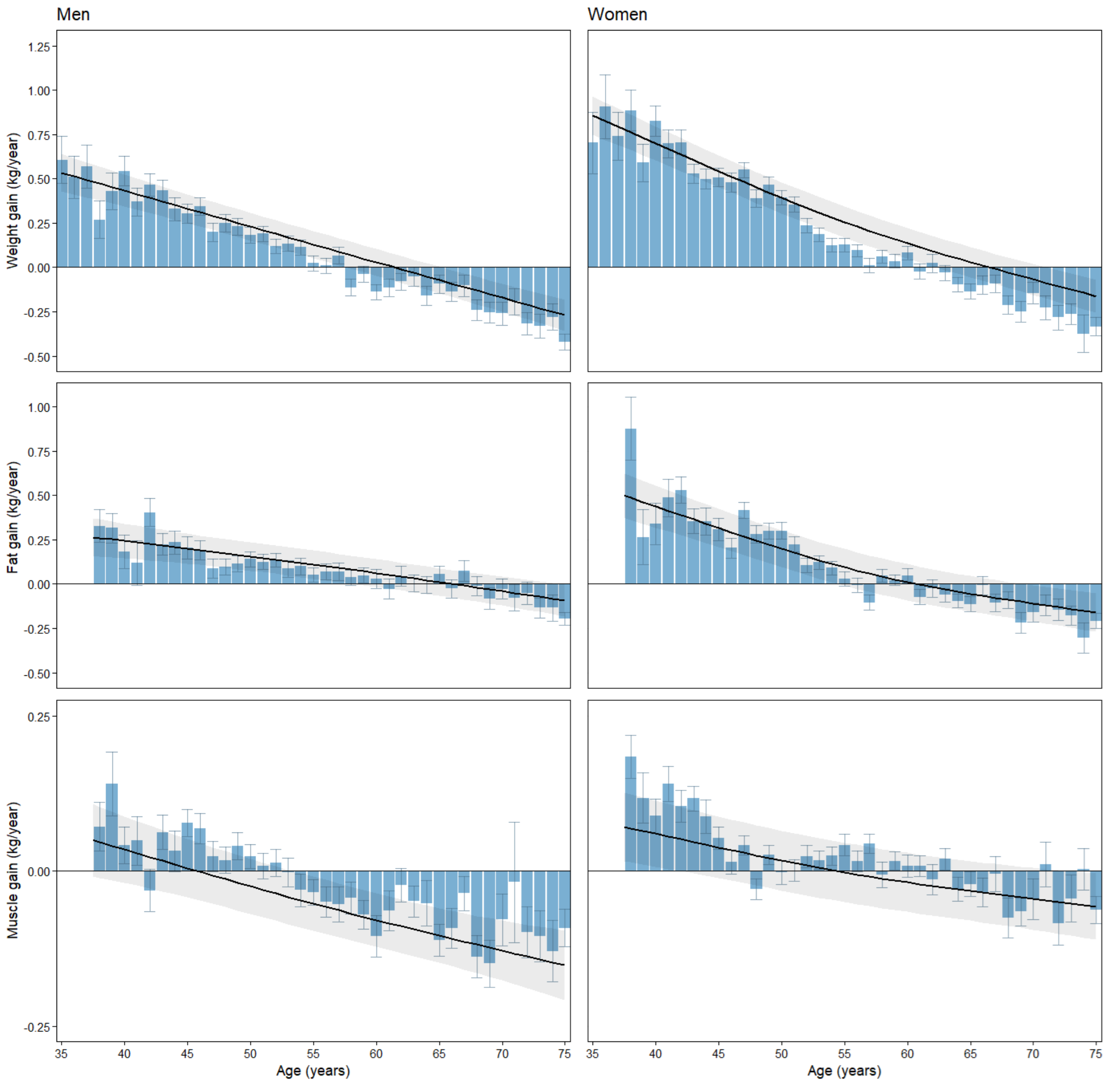

Supplementary Figure S2. Annual weight, fat, and muscle gain (kg/year) by age (years), stratified by sex. The upper, middle, and lower panels represent weight gain, fat gain, and muscle gain, respectively. Bars show the observed mean annual gain for each age, while the solid line represents the adjusted restricted cubic spline estimated using generalized estimating equations (GEE). Models were adjusted for race/skin color, education, income, BMI, and data collection period. Left: men; Right: women.

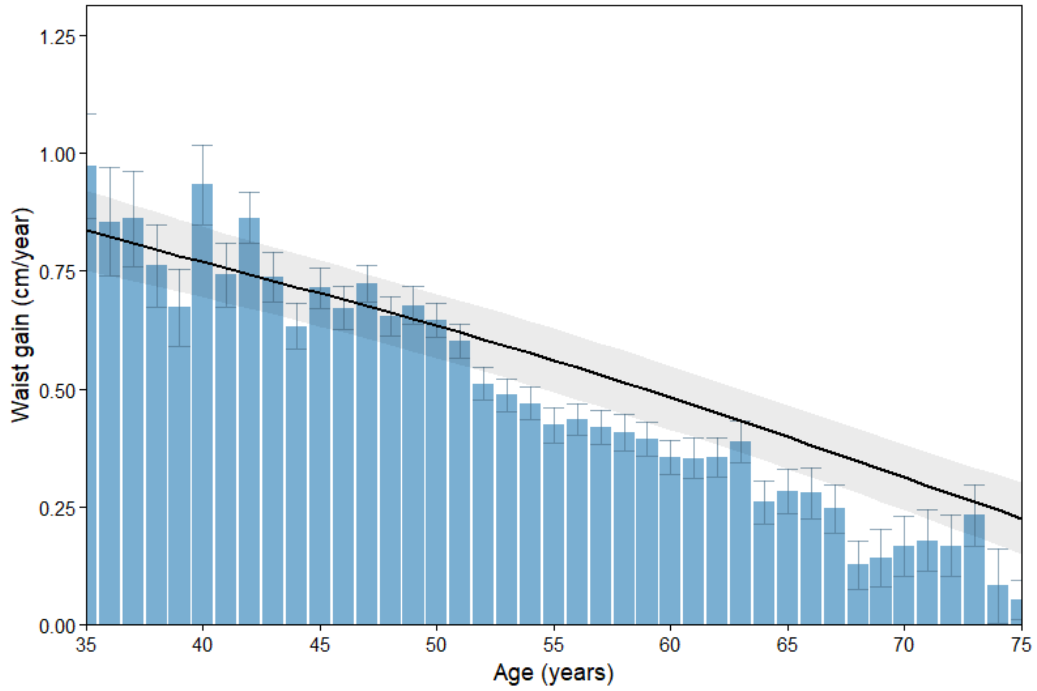

Supplementary Figure S3. Annual waist gain (cm/year) across age. Bars show the observed mean annual waist gain for each age, while the solid line represents the adjusted restricted cubic spline estimated using generalized estimating equations (GEE). Models were adjusted for sex, race/skin color, education, income, BMI, and data collection period.

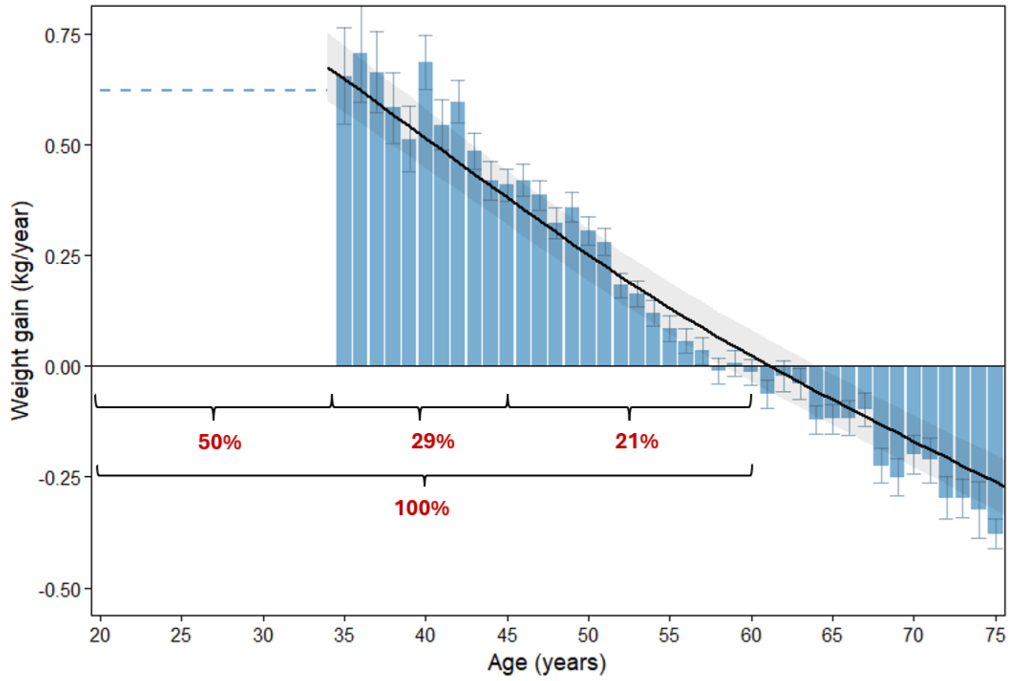

Supplementary Figure S4. Percentage of weight gain ascertained at different ages across the adult lifespan. ELSA-Brasil, 2008-2024.

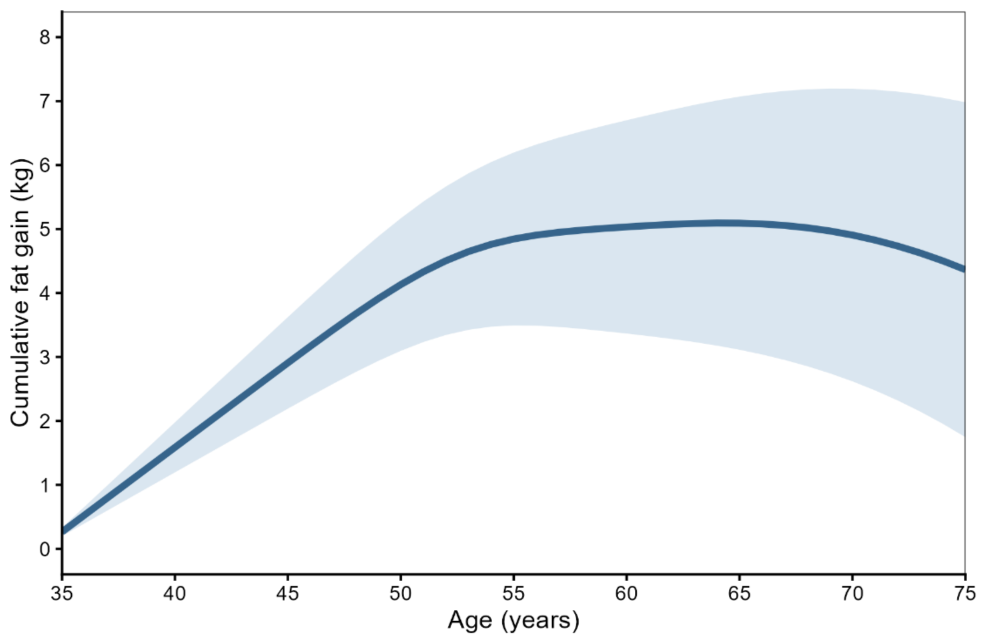

Supplementary Figure S5. Estimated cumulative fat mass gain (kg) across ages 35–75 years based on the adjusted restricted cubic spline model. The curve was estimated using generalized estimating equations (GEE) and adjusted for sex, race/skin color, education, income, BMI, and data collection period. ELSA-Brasil, 2008-2024.

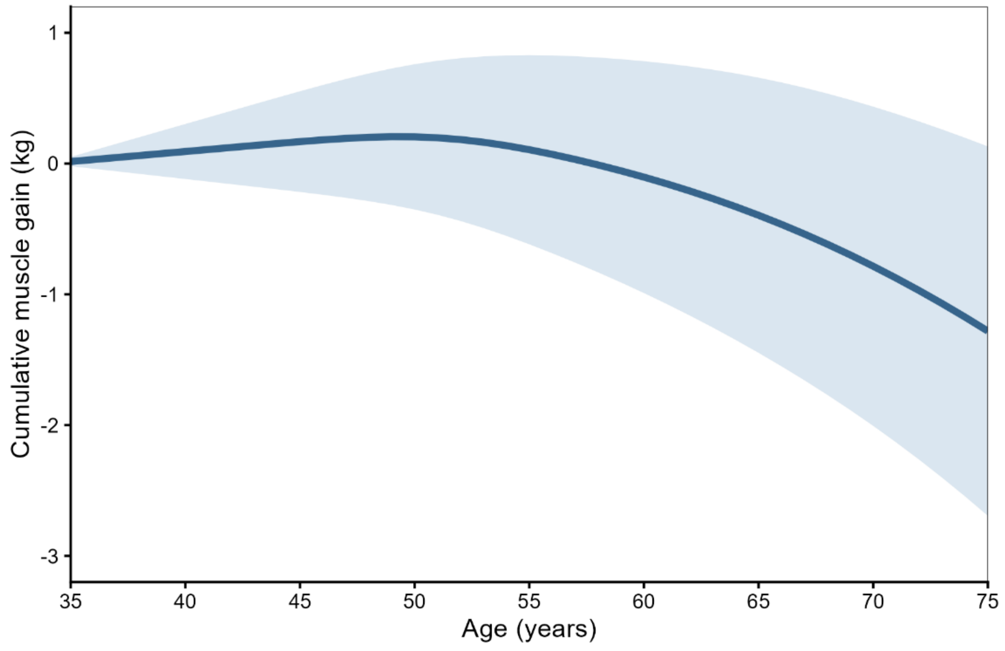

Supplementary Figure S6. Estimated cumulative muscle mass gain (kg) across ages 35–75 years based on the adjusted restricted cubic spline model. The curve was estimated using generalized estimating equations (GEE) and adjusted for sex, race/skin color, education, income, BMI, and data collection period. ELSA-Brasil, 2008-2024.

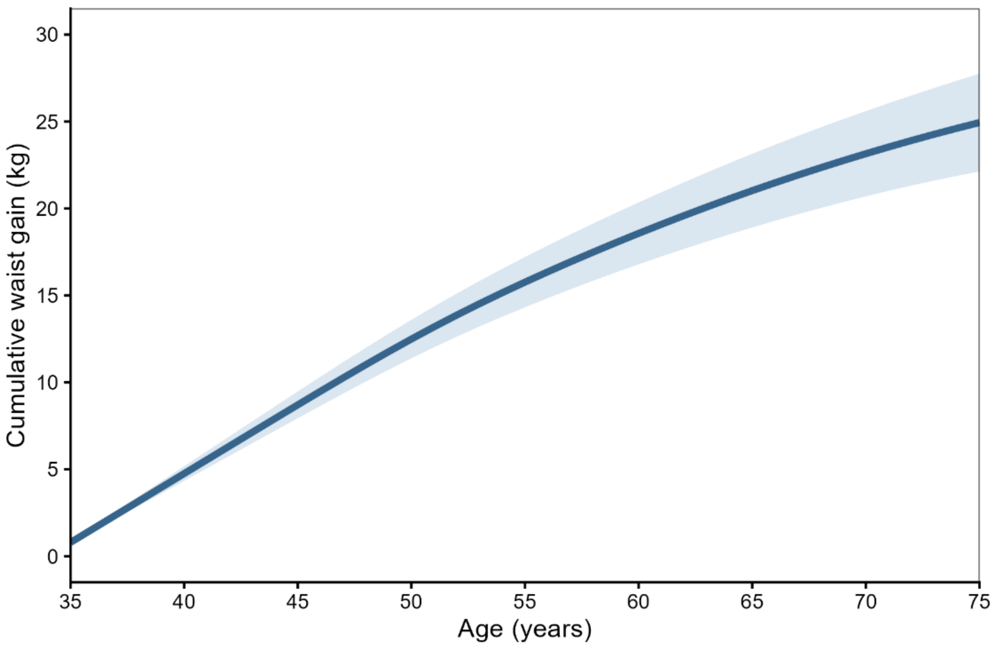

Supplementary Figure S7. Estimated cumulative waist circumference gain (cm) across ages 35–75 years based on the adjusted restricted cubic spline model. The curve was estimated using generalized estimating equations (GEE) and adjusted for sex, race/skin color, education, income, BMI, and data collection period. ELSA-Brasil, 2008-2024.
